# Isolation, biochemical characterization and enzymatic profiling of Plant Growth-Promoting Rhizobacteria associated with chilli (*Capsicum annuum* L.)

**DOI:** 10.1016/j.bbrep.2026.102559

**Published:** 2026-03-26

**Authors:** Pushpa Gehlot, Jyoti Yadav, Priya Soni, Tripta Jain

**Affiliations:** Department of Botany, Mohanlal Sukhadia University, Udaipur, Rajasthan, India

**Keywords:** PGPR, Chilli, Rhizosphere, Biofertilizer, Sustainable agriculture, Enzymatic activity, 16S rRNA, Phylogenetic analysis

## Abstract

The rhizosphere is a critical hotspot of plant-microbe interactions, where Plant Growth-Promoting Rhizobacteria (PGPR) play key roles in nutrient mobilization, growth promotion and stress tolerance. This study aimed to isolate and characterize PGPR from the rhizosphere of chilli (*Capsicum annuum* L.). Twenty-three morphologically distinct isolates were obtained and evaluated through morphological, biochemical, enzymatic and molecular approaches. Most isolates exhibited catalase and nitrate reduction activities, while carbohydrate utilization profiles revealed broader metabolic versatility in *Lysinibacillus macroides* compared to *Lysinibacillus fusiformis*. Enzymatic screening uncovered a high prevalence of protease, urease, amylase, cellulase and lipase production, key traits linked to nutrient cycling and rhizosphere colonization. Quantitative assessment of protease and lipase activities revealed significant inter-isolate variation, with isolates 4.1 and 2.B exhibiting comparatively higher enzyme indices. Pot tray validation showed that rhizobacterial inoculation enhanced seed germination and early seedling growth, with isolates 4.1 and 2.B performing best. Molecular identification confirmed isolates 4.1 and 2.B as *L. fusiformis* and *L. macroides*, respectively, supported by phylogenetic analysis. The dominance of diverse rhizobacterial strains and their hydrolytic enzyme activities reflects their ecological adaptability in semi-arid soils. These findings highlight *L. fusiformis* and *L. macroides* as promising biofertilizer candidates for chilli cultivation, offering eco-friendly alternatives to chemical fertilizers and contributing to sustainable, climate-resilient agriculture.

## Highlights

• Twenty-three chilli rhizobacteria were screened morphologically and biochemically.
• *L. fusiformis* and *L. macroides* exhibited strong PGPR and multi-enzyme activity.
• High metabolic versatility supported efficient nutrient mobilization.
• 16S rRNA analysis confirmed both isolates as potential biofertilizer candidates.

## 1 Introduction

One of the most important hotspots of biological interactions supporting plant health and productivity is the rhizosphere, the area of soil influenced by root secretions and associated microbial communities [1]. Among the various groups of microorganisms that live in the rhizosphere, Plant Growth-Promoting Rhizobacteria (PGPR) have attracted fascination due to their significant impact on disease prevention, nutrient dynamics and plant growth [2]. Both directly and indirectly, these bacteria support the growth of plants. While indirect mechanisms include pathogen antagonism through the production of antibiotics, siderophores, hydrogen cyanide and lytic enzymes, direct mechanisms include nitrogen fixation, phosphate solubilization and the synthesis of phytohormones like indole-3-acetic acid (IAA), gibberellins and cytokinins [3]. Restoring soil health, environmental safety and sustainability are becoming more and more important in the current agricultural paradigm [4]. A change to more sustainable methods has become necessary due to the detrimental consequences of excessive chemical fertilizer and pesticide use, which include soil degradation, biodiversity loss and groundwater contamination [5,6]. In this context, PGPR-based biofertilizers have emerged as eco-friendly alternatives for enhancing crop productivity, nutrient use efficiency and plant resilience [7].

India offers a rich environment for the study of native PGPR since it is a centre of diverse agro-ecological zones and a major crop producer. One of the most significant spice crops in the world is chilli (*Capsicum annuum* L.), which is extremely valuable both nutritionally and economically. Due to its high vitamin content, pungency and culinary versatility, chilli is grown all over the world [8]. However, nutrient deficiencies, degraded soil and disease susceptibility limit chilli production and frequently lead to reduced yields [9]. Since PGPR-based biofertilizers increase nutrient availability, strengthen plant defences and improve stress tolerance under field conditions, they offer an environmental friendly approach to addressing these challenges in chilli cultivation [10]. Plant growth promotion and stress resilience are strongly influenced by the enzymatic activities of rhizobacteria, which represent key functional traits of effective PGPR. Extracellular enzymes such as cellulases, proteases, amylases, urease and lipases are secreted by beneficial soil bacteria and contribute to organic matter decomposition and nutrient cycling in the rhizosphere [11]. These enzymatic processes release essential nutrients, including nitrogen, phosphorus and potassium, in plant-available forms, thereby improving root uptake and overall plant vigor [12]. In crops such as chilli, where nutrient limitations and soil-borne pathogens often constrain productivity, enzymatic activity provides a dual advantage by enhancing nutrient acquisition while mitigating biotic stress [13].

Recent advances in PGPR research have increasingly emphasized crop and strain-specific biofertilizer applications in Solanaceae crops, including chilli (*Capsicum annuum* L.). Studies have reported that biofertilizers based on *Bacillus* spp. and *Lysinibacillus* spp., applied either alone or in combination with reduced chemical fertilizer inputs, significantly enhance plant phosphorus and potassium uptake and improve soil nutrient status, supporting their role in sustainable nutrient management strategies [14]. These findings highlight the strong nutrient-mobilizing potential of *Lysinibacillus* spp. and underscore the growing need for strain-level functional validation rather than genus-level generalizations. Earlier physiological and molecular studies have established *Lysinibacillus* spp. as emblematic PGPR with diverse enzymatic capabilities linked to nutrient solubilization and plant growth promotion [15]. In addition, stress-mitigation functions relevant to chilli cultivation have been reported, where heavy metal-tolerant *Lysinibacillus* spp. alleviated cadmium and lead stress by enhancing root-associated growth and early seedling development [16]. More recently, indigenous microorganisms derived from fermented food systems, including belacan, have emerged as novel PGPR sources, with *Lysinibacillus fusiformis* and *Bacillus velezensis* shown to promote chilli growth through nutrient solubilization and auxin production, resulting in improved shoot development and chlorophyll content [14].

Despite these advances, most existing studies emphasize genus or species-level characterization, while strain-level functional differentiation within defined crop-soil systems remain insufficiently explored. In particular, information on chilli-associated *Lysinibacillus* isolates from semi-arid agroecosystems and their comparative enzymatic and metabolic profiles is limited. In this context, the present study aimed to isolate and identify PGPR from the chilli rhizosphere and to characterize their functional potential using morphological, biochemical, molecular and enzymatic approaches. By documenting the diversity and strain-specific functionality of chilli-associated rhizobacteria, this research seeks to support the development of effective, crop-specific microbial biofertilizers and contribute to sustainable agriculture through improved plant-microbe interactions.

## 2 Materials and methods

### 2.1 Collection of rhizospheric soil samples

Rhizosphere soil samples were collected from actively cultivated chilli fields located at the Rajasthan College of Agriculture (24°34′50.3″ N, 73°42′07.7″ E), Udaipur, Rajasthan, India. Soil samples were collected during the active vegetative growth stage of chilli (Rabi season). Rhizospheric soil was collected from a depth of 5-15 cm by gently uprooting chilli plants and collecting soil tightly adhering to the roots. Root-adhering soil was carefully excavated, collected in sterile polyethene bags, transported to the laboratory and stored at 4 °C for further analysis. The soil pH was measured using a digital pH meter and was found to be near neutral (pH 6.8-7.2). The experimental soil was loamy in texture, representative of commonly cultivated agricultural soils of the region.

### 2.2 Isolation of Rhizobacteria

Isolation of bacteria from the rhizospheric soil samples was carried out using the serial dilution technique [17]. One gram of air-dried and sieved soil sample was suspended in 9 mL of sterile distilled water and homogenized thoroughly. The suspension was subjected to serial dilution from 10^−1^ to 10^−6^. Aliquots of 100 μL from appropriate dilutions were spread evenly on solidified nutrient agar (NA) plates (HiMedia Laboratories Pvt. Ltd., India) using a sterile L-shaped spreader. The plates were incubated at 37 ± 2 °C under aerobic conditions for 48-72 h**,** and bacterial colonies were observed periodically.

### 2.3 Development of pure culture of isolates

Distinct colonies exhibiting diverse morphological characteristics were randomly selected and re-streaked on fresh nutrient agar plates to obtain pure cultures. The plates were incubated at 37 ± 2 °C for 48h under aerobic conditions. All cultures were sub-cultured at regular intervals to maintain viability and purity. Nutrient agar (NA) (HiMedia Laboratories Pvt. Ltd., India) was used for routine cultivation of bacterial isolates. For long-term preservation, pure cultures were stored in nutrient broth supplemented with 20% glycerol and maintained at **−**4°C.

### 2.4 Morphological characterization

Preliminary identification of bacterial isolates was performed based on colony morphology on nutrient agar. Cultural characteristics such as colony shape, pigmentation, margin, elevation and texture were recorded. After Gram staining, bacterial cell shape and morphology were examined under a light microscope (400× magnification). The gram staining procedure was conducted as per standard protocols [18] and isolates were categorized as gram-positive or gram-negative based on their staining reaction and cell morphology.

### 2.5 Biochemical characterization

Biochemical characterization provides critical insights into the metabolic properties and enzymatic activities of bacterial isolates, facilitating their identification at the genus level. A series of biochemical tests was employed in this study, following the standardized protocols outlined in Bergey's Manual of Determinative Bacteriology [19] and Cappuccino and Sherman [18]. All reagents were of analytical grade and controls were included where applicable. These tests provided preliminary taxonomic insights and guide for further molecular identification.

#### 2.5.1 Catalase test

The catalase test evaluates the ability of bacterial isolates to decompose hydrogen peroxide (H_2_O_2_) into water and oxygen, a key trait for differentiating between aerobic and facultative anaerobic organisms [20]. Few drops of fresh bacterial suspension culture were placed onto a clean glass slide and a drop of 3% H_2_O_2_ was added. Immediate effervescence due to oxygen release was interpreted as a positive reaction, indicating catalase enzyme presence.

#### 2.5.2 Indole production test

This test assesses the capacity of bacteria to degrade the amino acid tryptophan into indole using the enzyme tryptophanase [21]. Isolates were inoculated into sterile tryptone broth and incubated at 35 ± 2 °C for 48 h. After incubation, 0.5 mL of Kovac's reagent was added. The development of a cherry-red layer at the interface indicated a positive result, signifying indole production.

#### 2.5.3 Methyl red (MR) test

The MR test identifies bacteria capable of performing mixed acid fermentation of glucose, resulting in stable acidic end products [22]. Isolates were grown in MR-VP broth for 48 h at 37 °C. A few drops of methyl red indicator were added post-incubation. A persistent red colour indicated a positive MR test, suggesting stable acid production.

#### 2.5.4 Voges proskauer (VP) test

Complementary to the MR test, the VP test detects the production of neutral end products such as acetoin (acetylmethylcarbinol) from glucose fermentation via the butylene glycol pathway [22]. To 10 mL of 48 h old MR-VP culture, 6 mL of α-naphthol (5%) and 2 mL of 40% KOH were added. The development of a pink to red color within 30 min confirmed a positive result.

#### 2.5.5 Nitrate reduction test

The nitrate reduction test evaluates the ability of bacteria to use nitrate (NO_3_^−^) as a terminal electron acceptor under anaerobic conditions. Nitrate reductase reduces nitrate to nitrite, detected by the formation of a red diazonium complex after adding sulfanilic acid (Reagent A) and α-naphthylamine (Reagent B). If no color appears, zinc powder is added: a red color indicates unreduced nitrate (negative), while no color confirms complete reduction beyond nitrite (positive) [21]. Bacterial isolates were inoculated into sterile nitrate broth tubes and incubated at 35 ± 2 °C for 24-48 h. Post-incubation, 5 drops each of Reagent A and Reagent B were added. Color development was observed within 1-2 min.

#### 2.5.6 Sugar utilization test

The carbohydrate fermentation profiles of bacterial isolates were determined using the HiCarbo™ KB009 kit (HiMedia, India), which contains 3 parts. Part A, Part B and Part C. This kit comprises 35 dehydrated carbohydrates and four biochemical substrates in individual wells. Bacterial suspensions were prepared in nutrient broth. A 100 μL aliquot was inoculated into each well. The strips were incubated at 35 ± 2 °C for 24-48 h in a humid chamber. A colour change indicated fermentation. The sugar utilization profile was recorded as positive or negative and used for phenotypic characterization of the bacterial isolates [22].

All biochemical assays were conducted in triplicate and consistent results across replicates were considered valid. Negative and positive control strains were used to validate the accuracy of each test. The comprehensive phenotypic and biochemical profiles obtained were compared against standard taxonomic keys to support bacterial identification and variation.

## 3 Enzymatic activity

The isolated bacterial strains were screened for amylase, protease, lipase, urease and cellulase activity using the plate assay method.

### 3.1 Amylase production test

Amylase production test was performed as described by Aneja [17]. One loopful of 24 h old bacterial suspensions of each isolated strains were streaked on starch agar medium (SAM) separately. Plates were incubated at 37 °C for 48 h. After incubation, 1% iodine solution was flooded on inoculated petriplates for 5 min. Observations were recorded by the presence and absence of clear zone around the bacterial strains. The clear zone around the bacterial colonies showed a positive test for amylase production.

### 3.2 Proteinase production test

Proteinase production was determined as described by Aneja [17]. One loopful of a 12-h-old bacterial suspension of each isolate was spot-inoculated onto skim milk agar (SMA) plates and incubated at 37 °C for 48 h. Proteolytic activity was indicated by the formation of a clear halo zone around the bacterial colonies. The diameter of the halo zone and the colony was measured (in mm), and the proteolytic index was calculated as the ratio of the total diameter (colony + halo zone) to the colony diameter.

### 3.3 Lipase test

Lipase activity was assessed on tributyrin agar medium (g L^−1^) composed of peptone (5.0), beef extract (3.0), tributyrin (10 mL), and agar (15), adjusted to pH 6.5. Bacterial isolates were spot-inoculated and incubated at 37 °C for 48 h. Lipase production was evidenced by the appearance of a transparent zone surrounding the colonies. The diameter of the clearance zone was measured in millimetres, and lipolytic activity was expressed as a lipase index based on the ratio of the diameter (colony + halo zone) to colony diameter [23].

### 3.4 Urease test

Bacterial isolates were inoculated on Christensen's urea agar slants and incubated at 37 °C for 48 h. A pink color change indicated a positive urease reaction [24].

### 3.5 Cellulase test

Czapek's mineral salt agar media was prepared, inoculated and incubated for 48 h at 37 °C. After the growth of the colonies, plates were flooded with dye (congo red) for 15 min for staining and 1% NaCl for destaining for 15 min. After destaining, halo zone formation around the bacterial colonies was observed [25].

Based on the preliminary screening of 23 isolates, strains 4.1 and 2.B were selected for detailed characterization owing to their consistent expression of multiple hydrolytic enzymes, broader carbohydrate utilization profiles and overall functional performance in comparison to other isolates.

## 4 Molecular identification via 16S rRNA gene sequencing

The best screened isolates 4.1 and 2.B were sent to species-level identification. Genotyping was done at Biokart India Pvt. Ltd. Banglore. The plates were sealed with parafilm, packed along with ice pack were sent to Biokart India Pvt. Ltd. for isolation of bacterial genomic DNA, amplification of bacterial 16S rRNA using PCR and sequencing of the fragment.

### 4.1 Genomic DNA isolation

Genomic DNA was extracted from pure bacterial cultures using the XPLOREGEN gDNA Extraction Kit (Xploregen Discoveries Pvt. Ltd., Bengaluru, India) following the manufacturer's protocol. Briefly, 1 mL of XPLOREGEN gDNA Extraction Buffer™ 1 was added to the beaded vial, followed by the sample. The mixture was subjected to horizontal vortexing at maximum speed for 10 min. Subsequently, 300 μL of Buffer™ 2 was added, and the vial was again vortexed for 7 min. The lysate was centrifuged at 10,000 rpm for 3 min at room temperature, and 800 μL of the supernatant was transferred to a sterile 2 mL tube. To this, 200 μL of Buffer™ 3 was added, briefly vortexed (5 s) and centrifuged at 10,000 rpm for 2 min. The resulting supernatant (800 μL) was transferred to a fresh 2 mL sterile tube, followed by the addition of 1 mL Buffer™ 4 and a brief vortex.

Subsequently, 700 μL of the lysate was loaded onto a spin column and centrifuged at 10,000 rpm for 2 min. The process was repeated with the remaining lysate to ensure complete loading. After discarding the flow-through, the column was washed sequentially with 600 μL each of Buffer™ 5 and Buffer™ 6, centrifuging after each step at 10,000 rpm for 2 min. An additional centrifugation step of 5 min was performed to remove residual wash buffers. The spin column was then transferred to a sterile 1.5 mL microcentrifuge tube and 30 μL of Elution Buffer™ 7 was added directly to the centre of the membrane for elution. After centrifugation at 10,000 rpm for 5 min, the column was placed into a new sterile 1.5 mL tube for a second elution. The eluates were collected and stored at −20 °C for downstream molecular applications [26].

### 4.2 PCR amplification of 16S rRNA gene

The 16S rRNA gene was amplified using universal bacterial primers.

**16S Forward: GGATGAGCCCGCGGCCTA**
**16S Reverse: CGGTGTGTACAAGGCCCGG**

The 16S rRNA gene was amplified using 169 ng of genomic DNA as template in a 50 μL reaction volume. The reaction mixture comprised 1 μL of 10 pM 16S forward primer, 2 μL of 10 pM 16S reverse primer, 4 μL of dNTPs (2.5 mM each), 10 μL of 10 × Taq DNA polymerase assay buffer, 1 μL of Taq DNA polymerase (3 U/μL) and nuclease-free water to make up the final volume. The Taq Master Mix contained a high-fidelity DNA polymerase, 2.5 mM dNTPs, 3.2 mM MgCl_2_ and enzyme buffer [27].

PCR was carried out under the following thermal cycling conditions: initial denaturation at 94 °C for 3 min, followed by 30 cycles of denaturation at 94 °C for 1 min, annealing at 50 °C for 1 min and extension at 72 °C for 2 min. A final extension was performed at 72 °C for 7 min. The amplified products were stored at 4 °C until further analysis.

### 4.3 DNA sequencing and analysis

The purified PCR products were sequenced bi-directionally using Sanger sequencing technology. Sequence identification was carried out using the BLASTn algorithm against the NCBI GenBank databases. A similarity score of ≥97% was considered sufficient for genus-level identification and ≥99% for species-level identification.

**Submission to Public Database:** All high-quality 16S rRNA gene sequences were submitted to the NCBI GenBank database and accession numbers were obtained.

### 4.4 Construction of phylogenetic tree

A phylogenetic tree was constructed using the Neighbor-Joining (NJ) method, which is widely applied for constructing distance-based trees due to its computational efficiency and robustness. Evolutionary distances were computed using the Kimura 2-parameter (K2P) model, which corrects for multiple substitutions at the same site and differences in transition/transversion rates. Trees were visualized and exported using MEGA X and phylogenetic clusters were interpreted in the context of taxonomic affiliations.

## 5 Pot trial experiment for evaluating plant growth promotion in chilli

Pot trials were performed using chilli seed variety 'PUSA JWALA'. Seeds were surface-sterilized with 0.02% sodium hypochlorite for 2 min, and rinsed thoroughly in sterile distilled water [28]. Pot trials were conducted to evaluate the effectiveness of rhizobacterial strains for improving the growth of chilli plants. The sterilized seeds were inoculated with bacterial isolates for 30 min for seed priming as per Vidhyasekaran and Muthamilan [29], whereas uninoculated seeds were used as a control. Plastic small pots were filled with sterilized potting soil and five treated seeds were sown per pot. In the experiment soil were treated with 20 ml of rhizobacterial strains (10^−8^ cfu/ml) separately [30]. In negative control in place of inoculums sterilized water was used. Each treatment was replicated thrice. Chilli plants were harvested after 45 days of sowing and data were recorded regarding root-shoot length, no. of leaves emerged and no. of seed germinated.

## 6 Results

### 6.1 Isolation and morphological characterization of rhizospheric bacteria

A total of 23 morphologically distinct bacterial isolates were obtained from the rhizospheric soils of *Capsicum annuum* L. (chilli). The isolates exhibited diverse colony morphologies on nutrient agar, ranging from circular, mucoid, cream-colored colonies to irregular, dry and pigmented forms. While some Gram-negative cocci and rods were also seen, Gram staining showed that Gram-positive rods predominated (Table 1). These morphological features indicated taxonomic heterogeneity among the rhizospheric bacterial community.

**Table 1:** Phenotypic characteristics of different bacterial isolates. An asterisk (∗) indicates the isolate name.

S. No.	Bacterial Isolates	Colony Colour	Growth Rate	Morphology	Shape	Gram Staining
1.	4.1	Dark Cream	Fast	Rough	Rod	Gram positive
2.	2.b	Cream	Fast	Rough	Rod	Gram positive
3.	10.2	White	Moderate	Smooth	Cocci	Gram positive
4.	6.2.b	Bright Yellow	Very Slow	Smooth	Rod	Gram negative
5.	8.b	Cream	Fast	Rough	Rod	Gram positive
6.	8.e	Cream	Moderate	Rough	Cocci	Gram positive
7.	6.c	Dark Cream	Moderate	Rough	Rod	Gram positive
8.	1.1	Off- white	Fast	Rough	Cocci	Gram negative
9.	6.1.b	Off- white	Moderate	Smooth	Rod	Gram positive
10	8.c	Cream	Moderate	Rough	Cocci	Gram negative
11.	2.1	White	Moderate	Rough	Rod	Gram negative
12.	2.2	Off- white	Moderate	Rough	Rod	Gram positive
13.	8.d	Off- white	Fast	Rough	Cocci	Gram negative
14.	7.1	White	Fast	Smooth	Rod	Gram positive
15.	TPG5	Cream	Fast	Smooth	Rod	Gram positive
16.	TPG8	Cream	Fast	Rough	Rod	Gram negative
17.	TPG6	Dark Cream	Slow	Smooth	Rod	Gram negative
18.	TPG7	Light Yellow	Slow	Rough	Cocci	Gram positive
19.	TPG1	Light Yellow	Slow	Smooth	Rod	Gram negative
20.	TPG4	Off-white	Slow	Smooth	Rod	Gram positive
21.	1.P	Light yellow	Slow	Rough	Rod	Gram negative
22.	1.B	White	Fast	Smooth	Rod	Gram positive
23.	2.1.1∗	Off white	Slow	Rough	Rod	Gram positive

### 6.2 Biochemical profiling of isolates

**Fig. 1:**
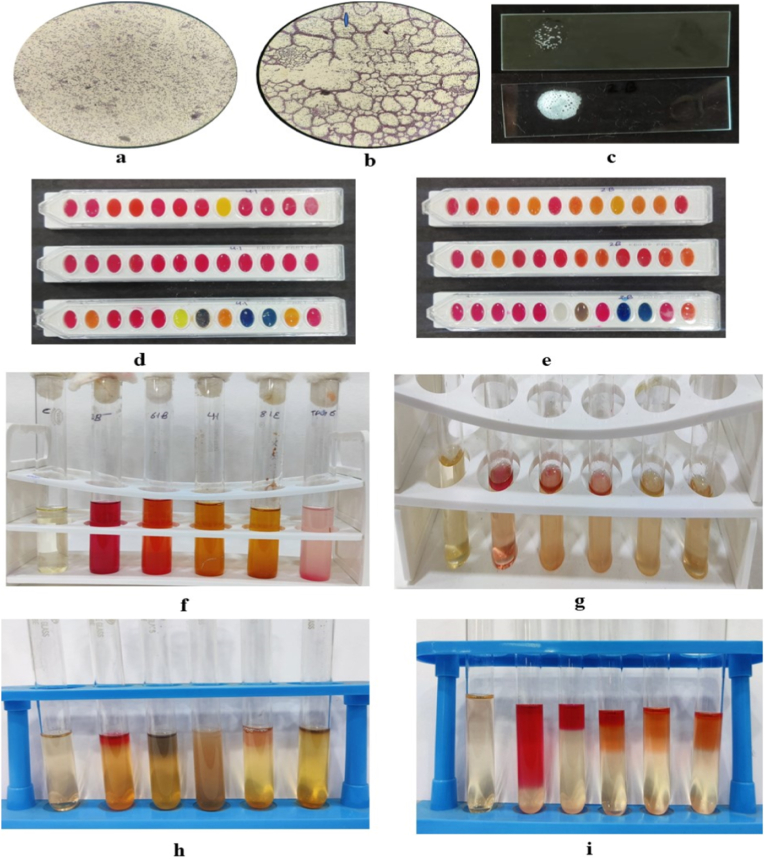
Biochemical and morphological characterization of screened rhizobacterial isolates. **(a)** Gram staining of isolate 4.1 (*Lysinibacillus fusiformis*). **(b)** Gram staining of isolate 2.B (*Lysinibacillus macroides*). **(c)** Catalase activity of isolates 4.1 (*Lysinibacillus fusiformis*) and 2.B (*Lysinibacillus macroides*). **(d**–**e)** Carbohydrate utilization profiles determined using HiCarbo™ kits (Parts A, B, and C). **(f)** Nitrate reduction test. **(g)** Indole production test. **(h)** Voges-Proskauer (VP) test. **(i)** Methyl red (MR) test.

**Fig. 2:**
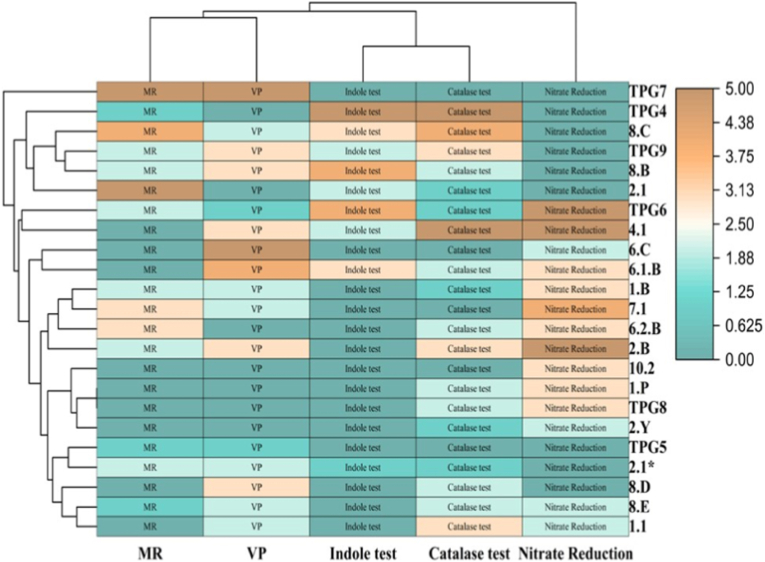
Hierarchical clustering heatmap of biochemical traits of chilli rhizospheric isolates. MR, VP, Indole, Catalase, and Nitrate Reduction tests are shown. Color intensity indicates relative activity from low to high. Row dendrograms represent similarity-based clustering, highlighting biochemical diversity among isolates.

All isolates were subjected to a set of biochemical tests including catalase, nitrate reduction, indole production, methyl red, voges-proskauer test and carbohydrate utilization assays (Fig. 1). Approximately 30% were indole positive, while 60% were positive for Methyl red and voges-proskauer test. Catalase activity was observed in 80% of the isolates. The majority of isolates tested positive for nitrate reduction. The carbohydrate utilization analysis suggests that *L. macroides* possesses a wider carbohydrate utilization spectrum than *L. fusiformis*, reflecting potential ecological adaptability in diverse rhizospheric environments. The carbohydrate assimilation profiles obtained are summarized in Table 2. These traits suggested the presence of metabolically diverse and functionally active bacterial communities in the rhizosphere. Fig. 1, Fig. 2, present the morphological and biochemical features of the screened bacterial isolates.

**Table 2:** Carbohydrate utilization test of *Lysinibacillus fusiformis* and *Lysinibacillus macroides* (Hi-Carbo, 35 substrates).

S. No.	Carbohydrate/Substrate	*Lysinibacillus fusiformis* (Isolate 4.1)	*Lysinibacillus macroides* (Isolate 2.b)
1	Lactose	-	-
2	Xylose	+	-
3	Maltose	+	+
4	Fructose	+	+
5	Dextrose (Glucose)	+	+
6	Galactose	+	+
7	Raffinose	-	-
8	Trehalose	+	+
9	Melibiose	-	-
10	Sucrose	+	+
11	l-Arabinose	+	-
12	Mannose	+	+
13	Inulin	-	-
14	Sodium gluconate	-	-
15	Glycerol	+	+ (weak)
16	Salicin	+	+
17	Dulcitol	-	-
18	Inositol	-	-
19	Sorbitol	-	-
20	Mannitol	+	+
21	Adonitol	-	-
22	Arabitol	-	-
23	Erythritol	-	-
24	α-Methyl-D-glucoside	+	+
25	Rhamnose	+	-
26	Cellobiose	+	+
27	Melezitose	-	-
28	α-Methyl-d-mannoside	-	-
29	Xylitol	-	-
30	ONPG (β-galactosidase)	-	-
31	Esculin	+	+ (weak)
32	d-Arabinose	+	-
33	Citrate	+ (weak)	+ (weak)
34	Malonate	-	-
35	Sorbose	-	-

### 6.3 Enzymatic activity

#### 6.3.1 Amylase production test

On qualitative screening of amylase production activity, development of colourless zone around the bacterial strain on SAM indicates hydrolysis of starch in the vicinity of bacteria colony by amylase production. Out of 23 rhizobacterial isolates 11 isolates were found positive and 12 were found negative for amylase production as shown in Fig. 5, Fig. 6.

#### 6.3.2 Proteinase production test

**Fig. 3:**
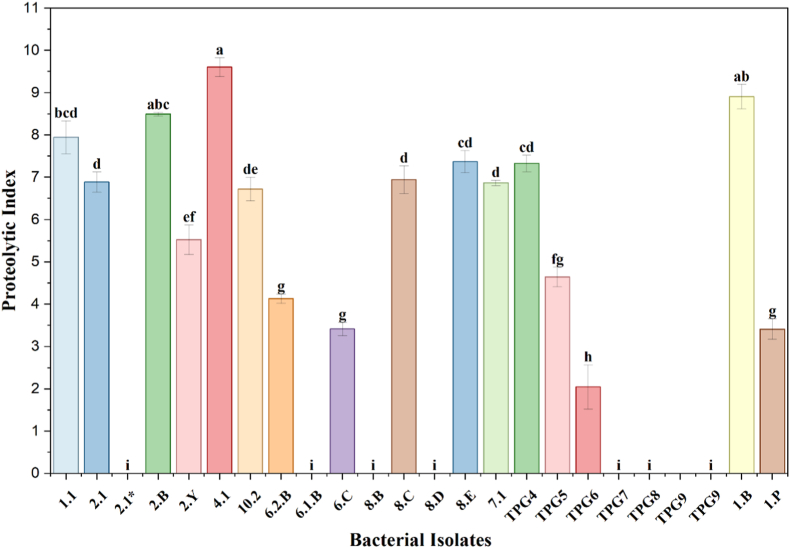
Proteolytic index of bacterial isolates. Values are mean ± SD. Different letters above bars denote significant differences (*p* ≤ 0.05).

Qualitative screening for protease activity revealed the development of clear zones surrounding bacterial colonies on skim milk agar (SMA), resulting from the hydrolysis of casein into soluble nitrogenous compounds, thereby confirming the proteolytic potential of the strains. Out of 23 rhizobacterial isolates 16 were found positive and 7 were found negative for proteinase production as shown in Fig. 6. Proteolytic index analysis further revealed significant inter-isolate variation, with isolates 4.1, 1.B, and 2.B exhibiting the highest protease activity, while several others demonstrated moderate to weak activity (Fig. 3).

#### 6.3.3 Lipase test

**Fig. 4:**
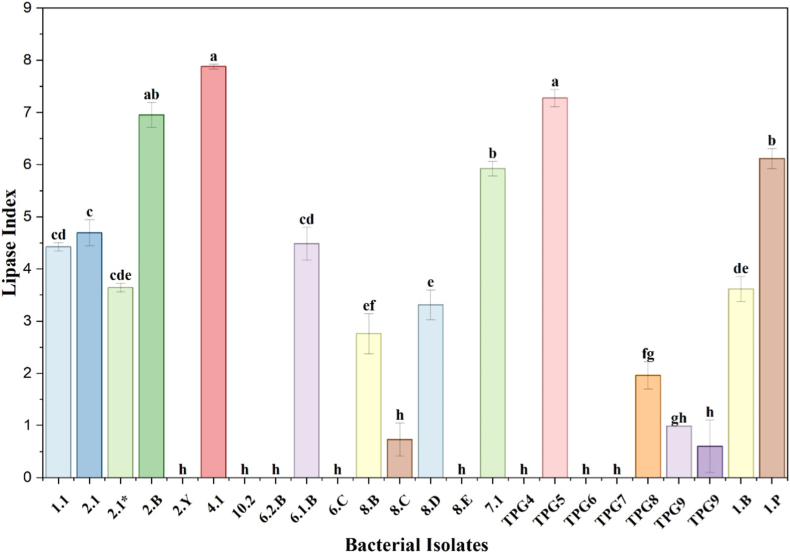
Lipase index of bacterial isolates. Values represent mean ± SD. Different letters above bars denote significant differences (*p* ≤ 0.05).

The lipolytic activity of the rhizobacterial isolates was assessed on tributyrin agar, where hydrolysis of tributyrin by extracellular lipase resulted in the formation of clear halo zones around bacterial colonies. Marked variation in lipase activity was observed among the isolates, as reflected by differences in lipase index values (Fig. 4). Of the 23 isolates screened, 15 exhibited positive lipase activity, while 8 isolates showed no detectable activity (Fig. 6). Several isolates, notably 2.B, 4.1, 1.1, 2.1, and TPG5, displayed comparatively higher lipase indices, indicating strong lipolytic potential, whereas others showed moderate to low activity. These results demonstrate isolate-specific variability in lipase production, highlighting the functional diversity of chilli-associated PGPR.

**Fig. 5:**
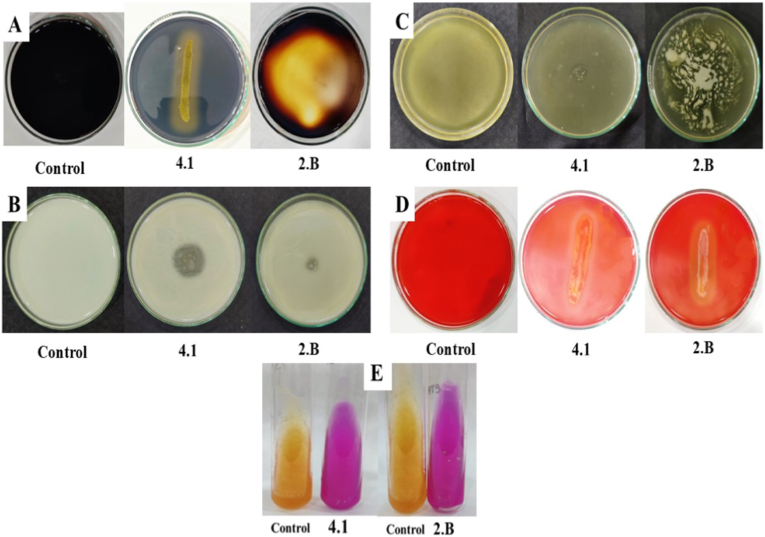
Enzymatic activities of rhizobacterial isolates. (A) Amylase, (B) protease, (C) lipase, (D) cellulase and (E) urease activities exhibited by bacterial isolates 4.1 (*Lysinibacillus fusiformis*) and 2.B (*Lysinibacillus macroides*) in comparison with the uninoculated control.

**Fig. 6:**
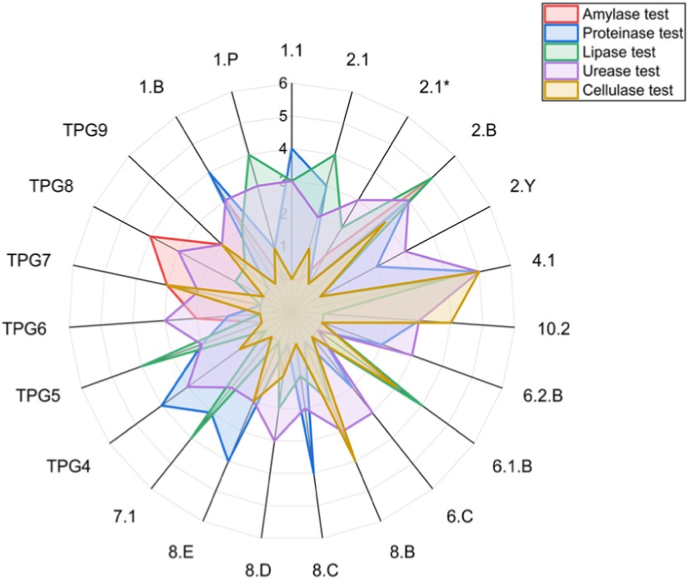
Radar plot of enzymatic activity of bacterial isolates.

#### 6.3.4 Urease test

Urease activity was assessed using Christensen's urea agar medium. A distinct color change from yellow to pink indicated positive urease production. Out of 23 rhizobacterial isolates 22 were found positive and 1 isolate was found negative for urease test as shown in radar plot (Fig. 6).

#### 6.3.5 Cellulase test

Cellulase activity of the isolates was confirmed on CMC agar, where hydrolysis zones appeared after Congo red staining. Out of 23 rhizobacterial isolates 12 were found positive and 11 isolate was found negative for cellulase test as shown in radar plot (Fig. 6). These results indicate that specific PGPR isolates possess significant cellulase activity, potentially enhancing rhizosphere colonization and organic matter degradation.

Among the 23 rhizobacterial isolates screened, strains 4.1 and 2.B were prioritized for further molecular and functional analysis based on their superior multi-enzyme activity and metabolic versatility (Table 3, Fig. 5, Fig. 6).

**Table 3:** Qualitative enzymatic and biochemical characterization of chilli rhizosphere bacterial isolates. Enzyme activities were assessed by plate assays and expressed semi-quantitatively (–, absent; + to +++++, increasing activity). Biochemical traits (MR, VP, nitrate reduction, indole, and catalase) were recorded as positive (+) or negative (−).

S. No.	Bacterial Isolates	Amylase test	Proteinase test	Lipase test	Urease test	Cellulase test	MR	VP	Nitrate Reduction	Indole test	Catalase test
1	1.1	-	++++	+++	+++	-	-	++	++	-	+
2	2.1	-	+++	++++	++	+	-	-	-	++	+
3	2.1∗	+	-	++	+++	-	++	++	-	+	+
4	2.B	+++++	++++	+++++	++++	+++	++	+++	+++++	-	+++
5	2.Y	-	++	-	+++	-	-	-	++	-	+
6	4.1	++++	+++++	++++	++++	+++++	-	+++	+++++	-	++
7	10.2	-	+++	-	+++	++++	-	-	+++	-	-
8	6.2.B	-	++	-	+++	-	-	-	+++	-	++
9	6.1.B	-	-	++++	-	+++	-	-	+++	-	++
10	6.C	-	++	-	+++	-	-	++	++	-	-
11	8.B	++++	-	++	+++	++++	++	+++	-	++++	++
12	8.C	-	++++	+	++	-	+	++	-	+++	+
13	8.D	+	-	++	+++	+	-	+++	-	-	++
14	8.E	-	++++	-	++	++	+	++	++	-	++
15	7.1	-	+++	++++	++	-	-	++	++++	-	-
16	TPG4	+	++++	-	+++	+	+	-	-	++	+++
17	TPG5	-	++	++++	++	-	+	+	-	-	-
18	TPG6	++	+	-	+++	-	++	+	++	++++	+
19	TPG7	+++	-	-	++	+++	+++	++++	-	-	-
20	TPG8	++++	-	+	+++	-	-	-	-	-	++
21	TPG9	++	-	+	++	++	++	+++	-	++	+
22	1.B	+++	++++	++	+++	-	++	++	+++	-	+
23	1.P	-	+	++++	+++	+	-	-	+++	-	++

### 6.4 Molecular identification

**Fig. 7:**
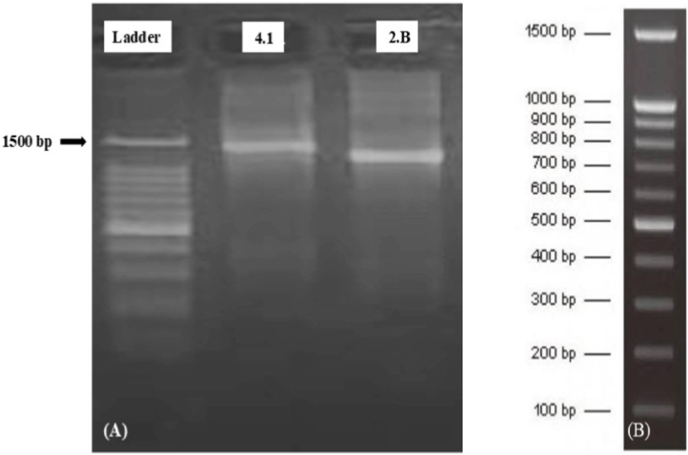
Agarose gel electrophoresis of PCR-amplified 16S rRNA gene fragments from selected rhizobacterial isolates. Lane L: 100 bp DNA ladder; lanes 1 and 2 represent bacterial isolates 4.1 and 2.B, respectively, showing amplification products of approximately ∼1500 bp, corresponding to the expected size of the bacterial 16S rRNA gene. Panel (B) shows the DNA ladder used for size reference.

The best screened isolates, 4.1 and 2.B, were sent for species-level identification. Using MEGA software, the isolates were identified as *Lysinibacillus fusiformis* CHL2 and *Lysinibacillus macroides* RH12 with the NCBI GenBank accession number PX482183 and PX482185 respectively. Agarose gel image of PCR-amplified 16S rRNA products from screened rhizobacterial isolates is shown in Fig. 7.

### 6.5 Phylogenetic analysis

The species-level confirmation of isolated rhizospheric strains had been performed on the basis of 16S r RNA gene sequence analysis and their sequence has been deposited in NCBI for accession number of the strains. The sequence analysis revealed the species level of strains and confirmed 4.1 as *Lysinibacillus fusiformis* strain CHL2 (Accession no.: PX482183) and 2.B as *Lysinibacillus macroides* strain RH12 (Accession no.: PX482185), on the basis of BLAST analysis. Based on their morphological and biochemical traits, the isolate 1.P and 1.B were identified as *Pseudomonas* sp. and *Bacillus* sp. respectively. *Bacillus* is the dominant group in the rhizosphere of Chilli.

**Fig. 8:**
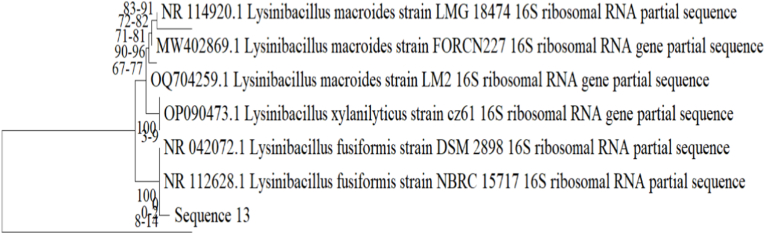
Phylogenetic tree for 4.1 (*Lysinibacillus fusiformis*) isolate.

**Fig. 9:**
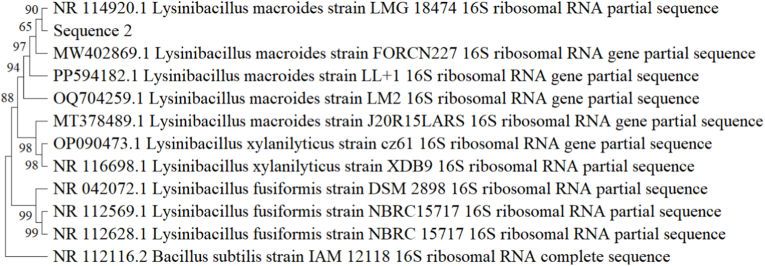
Phylogenetic tree for 2.B (*Lysinibacillus macroides*) isolate.

Phylogenetic tree of isolate 4.1 and 2.B based on its 16S rRNA Sequencing is given in Fig. 8, Fig. 9.

### 6.6 Effect of rhizobacterial inoculation on early plant growth

**Fig. 10:**
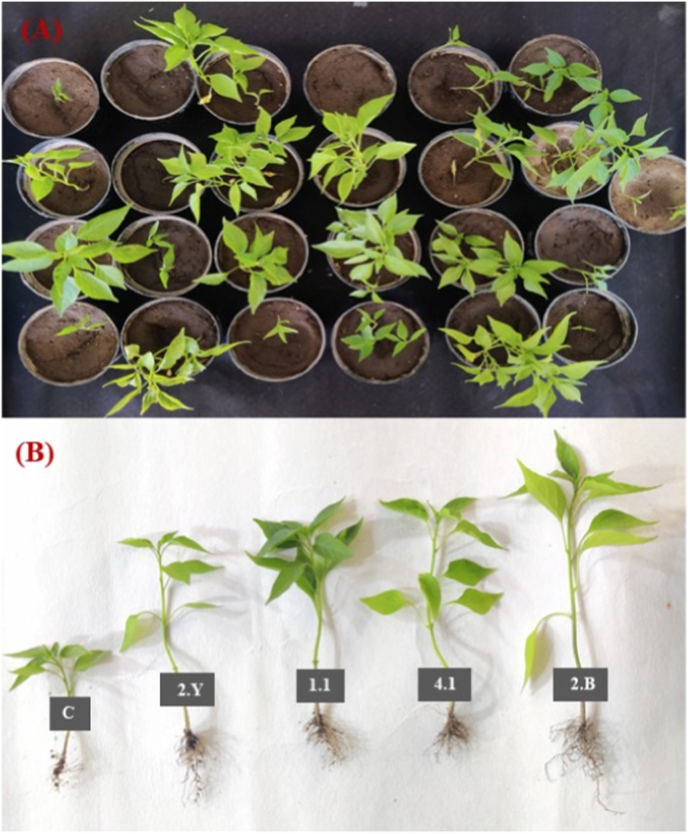
Pot trial showing the effect of rhizobacterial inoculation on chilli growth. (A) Chilli seedlings grown under different bacterial treatments. (B) Representative seedlings of isolates 2.Y, 1.1, 4.1 and 2.B, which exhibited enhanced shoot and root growth compared with the uninoculated control (C).

The pot tray experiment demonstrated clear differences in seed germination and early seedling growth among the rhizobacterial treatments (Fig. 10). Several isolates enhanced seed germination, whereas isolate 2.1 showed no germination. The highest germination (five seeds per pot) was observed with isolates 2.B and 4.1, followed by moderate germination in most other treatments.

**Fig. 11:**
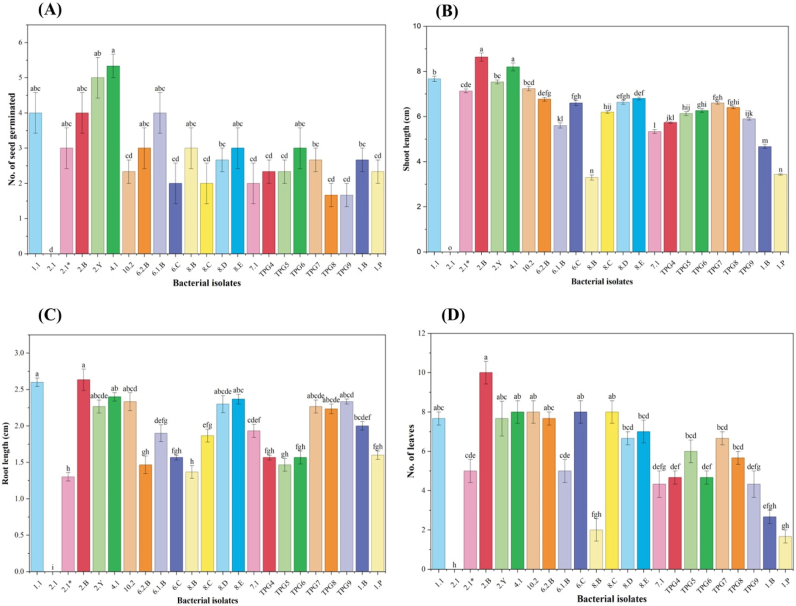
Effect of different rhizobacterial isolates on early growth parameters of chilli seedlings under pot tray conditions. (A) Number of seeds germinated, (B) shoot length (cm), (C) root length (cm), and (D) number of leaves recorded after inoculation with individual bacterial isolates. Bars represent mean values ± standard error. Different lowercase letters above bars indicate statistically significant differences among treatments at *p* ≤ 0.05 according to one-way ANOVA followed by a post hoc multiple comparison test.

Root and shoot growth parameters differed significantly among the rhizobacterial treatments (Fig. 11). Seedlings inoculated with isolate 2.B recorded the highest root length (≈2.9 cm), followed by 4.1 (≈2.5 cm), whereas several other isolates exhibited moderate root elongation. Shoot length was also significantly enhanced by isolates 2.B (≈8.9 cm) and 4.1 (≈8.2 cm) compared with the remaining treatments. Leaf development showed a similar response pattern. The maximum number of leaves was observed in seedlings treated with isolate 2.B (≈10 leaves), followed by 4.1 (≈9 leaves), while other isolates produced intermediate values. Overall, isolates 2.B and 4.1 consistently exhibited superior performance across all measured growth parameters under controlled pot conditions.

## 7 Discussion

The present study provides a detailed characterization of cultivable rhizobacterial isolates associated with the chilli rhizosphere, revealing substantial phenotypic and functional diversity. Variations in colony morphology, gram reaction, biochemical traits, carbohydrate utilization patterns, and enzymatic activities reflect the heterogeneous nature of the rhizosphere, where root exudates, nutrient availability, and micro-niche differentiation shape microbial populations. The integration of classical microbiological approaches with 16S rRNA gene sequencing strengthened taxonomic resolution and confirmed *Lysinibacillus fusiformis* and *Lysinibacillus macroides* as prominent members of the isolated PGPR community. The selection of strains 4.1 and 2.B for detailed analysis was guided by their overall functional performance across multiple screening assays, rather than by any single trait.

Both *L. fusiformis* and *L. macroides* exhibited multiple hydrolytic enzyme activities, including protease, lipase, urease, amylase and cellulase, indicating broad metabolic potential. Differences in the enzymatic profiles between these two species may reflect functional specialization rather than differences in overall ecological fitness. For example, cellulase and amylase production may facilitate the utilization of plant-derived polysaccharides released through root exudation or residue turnover, whereas protease and urease activities are associated with organic nitrogen mineralization [31]. Such enzymatic traits may enhance rhizosphere competence by improving access to localized nutrient pools, thereby supporting bacterial persistence in competitive root-associated environments [32].

Importantly, selected enzymatic traits were further quantified using enzyme index-based measurements. Proteolytic and lipolytic activities showed significant inter-isolate variation, with isolates 4.1 and 2.B exhibiting significantly higher proteolytic and lipase indices compared to other isolates (Fig. 3, Fig. 4). These quantitative differences, supported by statistical analysis, indicate strain-specific variation in extracellular enzyme production and strengthen the functional interpretation beyond qualitative screening alone.

However, the detection of enzymatic activity under *in vitro* conditions should be interpreted as an indication of functional potential rather than direct evidence of ecological adaptation or fitness in semi-arid soils. Enzyme expression and activity in natural soil systems are strongly modulated by physicochemical factors such as organic matter content and nutrient status, which were not assessed in the present study. In the absence of detailed soil physicochemical characterization, any direct linkage between enzymatic capacity and adaptation to semi-arid conditions must therefore be considered provisional. Accordingly, enzymatic versatility is more appropriately viewed as a contributory trait that may support rhizosphere colonization, rather than a definitive indicator of environmental adaptation.

To address this limitation and provide functional validation, a pot tray experiment was conducted to evaluate the effect of rhizobacterial inoculation on early plant growth. The enhanced seed germination, root and shoot elongation and increased leaf number observed in treatments inoculated with isolates 2.B and 4.1 (Fig. 10, Fig. 11) provide direct biological evidence supporting their plant growth-promoting activity. The consistency between high enzymatic activity indices and improved plant growth responses suggests that enzymatic versatility may contribute to early-stage plant establishment under controlled conditions.

From an agronomic perspective, the coexistence of multiple enzymatic functions in *L. fusiformis* and *L. macroides* suggests their potential involvement in nutrient mobilization and rhizosphere nutrient cycling, processes that may indirectly promote plant growth [33]. These observations are consistent with previous reports describing *Lysinibacillus* species as metabolically versatile PGPR [34]. Nevertheless, future studies integrating soil physicochemical analyses, and quantitative enzyme assays are required to validate the ecological relevance and functional performance of these isolates under field conditions, particularly in semi-arid agroecosystems. Such integrative approaches will be essential to substantiate their suitability as biofertilizer candidates for sustainable chilli cultivation.

## 8 Conclusion

This study reports the isolation and characterization of 23 cultivable rhizobacterial strains from the chilli rhizosphere based on morphological, biochemical, and enzymatic traits. Screening results indicated that several isolates expressed multiple plant growth-promoting attributes, including urease, protease, lipase, amylase, and cellulase activities. Among these, *Lysinibacillus fusiformis* and *L. macroides* demonstrated relatively broad carbohydrate utilization profiles and multiple enzymatic activities, suggesting functional versatility relevant to rhizosphere processes such as nutrient transformation. Quantitative assessment of protease and lipase activities revealed significant inter-isolate variation, with isolates 4.1 and 2.B exhibiting comparatively higher enzyme indices, thereby strengthening the functional interpretation of enzymatic screening beyond qualitative inference.

Importantly, the plant growth-promoting potential inferred from *in vitro* screening was further supported by a pot tray experiment conducted under controlled conditions Rhizobacterial inoculation resulted in statistically supported improvements in seed germination and early seedling growth parameters, with isolates 2.B and 4.1 consistently outperforming other treatments. These isolates promoted higher seed germination, enhanced root and shoot elongation, and increased leaf number, providing direct biological validation of their functional relevance during early plant establishment.

Although species of the genus *Lysinibacillus* have previously been reported as rhizosphere-associated bacteria with hydrolytic enzyme activities, the present study contributes a strain-level, crop-specific functional assessment of chilli-associated isolates under semi-arid conditions. The comparative enzymatic and metabolic profiling highlights context-dependent functional variability, rather than genus-level novelty. This study has certain limitations, as functional traits were assessed mainly through qualitative *in vitro* assays, which indicate potential activity but may not fully reflect performance under natural soil conditions. The lack of soil physicochemical characterization and field-level validation further limits inference on ecological fitness and long-term plant growth promotion. Nevertheless, the identified isolates represent promising biofertilizer candidates for chilli cultivation. Future studies should incorporate quantitative enzyme assays, soil analyses, field evaluations, formulation development, and investigations of synergistic interactions and genetic determinants to enable effective and sustainable agricultural application.

## Impact statement

This study is the first to report the isolation and comprehensive characterization of *Lysinibacillus fusiformis* and *Lysinibacillus macroides* from the chilli rhizosphere, revealing their unique biochemical and enzymatic traits that highlight their potential as crop-specific biofertilizers for semi-arid agriculture.

## CRediT authorship contribution statement

**Pushpa Gehlot:** Conceptualization, Data curation, Formal analysis, Methodology, Validation, Writing – original draft. **Jyoti Yadav:** Writing – review & editing. **Priya Soni:** Visualization. **Tripta Jain:** Supervision.

## Declaration of competing interest

On behalf of all authors, the corresponding author states that there is no conflict of interest.

## Data Availability

The dataset used and/or analysed during the current study available from the corresponding author on reasonable request.
